# Supplementary material for: Surveillance to maintain the sensitivity of genotype-based antibiotic resistance diagnostics
Source: PLoS Biol. 2019 Nov 12;17(11):e3000547. doi: 10.1371/journal.pbio.3000547 (PMC6874359; doi:10.1371/journal.pbio.3000547)
Supplement: Attachment — Submitted filename: phenotypic_sampling_response_to_reviewers_9-16.docx [file pbio.3000547.s001.docx]

We thank the reviewers for their constructive feedback, and we are grateful for the opportunity to revise our manuscript. The reviewers’ comments have led to substantial improvements in the interpretability of the results and, we hope, the manuscript’s clarity.

Reviewer #1: 

**Antibiotic resistance evolution is a significant problem and one way that is currently pursued to tackle the problem is the development of rapid diagnostics. These allow to not only identify the infectious agent, but more importantly the resistance profile and often mutation. A fundamental problem with this, as basically with almost all current approaches to tackle resistance, is that evolutionary change is an ongoing process and this fact is ignored. The paper presented here provides a tool to address this issue: it shows a way to estimate how much testing is required to detect newly evolved resistant variants, given the limits of current tests. As such, this is an interesting and worthwhile approach.

Overall, the paper is well written. I find though, that it lacks in detail and could also be clearer, given that the message is partly addressed at medical professionals with little time to read and digest.** 

**Lines 48-49. It is no really clear where these data are coming from. In the section on lines 58-73, two publications and NCBI accessions are cited, but it is mostly not clear to which panel in figure 1 they refer. Please clarify the source of the data for each panel of figure 1.**

We have now clarified the sources of the data (including pointers to figure panels) in Table 1. **Figure 1: I think it would be easier to read if fractional incidence and sensitivity were presented on separate panels. This would, especially in panels D and E make it much easier to see the differences between the dashed lines, which contain the main information. Also, I would suggest to briefly explain fractional incidence and sensitivity in the figure legend, to allow readers to understand the figure without going back to the text.**

We have now plotted fractional incidence and sensitivity in separate panels in Figure 1 and have added definitions for fractional incidence and sensitivity to the figure legend. **Lines 82 – 97. The approach is nice and simple. Yet, to reach a wider readership, it could be better explained. First, I would suggest making figure 1F a separate figure with a more informative caption. Also, I would suggest to explain, given the equation, how you arrived at the figures in lines 88 and following, as some readers will not spend much time on the equations.**

Figure 1F has now been incorporated into Figure 2, and the legend has been expanded. We have also clarified how the figures in lines 99-104 (formerly lines 88-92) were calculated.

**Another aspect worth considering here would be that, as also shown in figure 1, different variants might emerge. A brief discussion whether or not that matters would be useful.**

Thank you for this comment. Given that our model for calculating sampling fraction as a function of the total number of cases with the novel variant prior to detection is based on very few assumptions and variables (*e.g.*, it is not dependent on variant growth rate), it should be generalizable to any variant. We have now explicitly stated this in the manuscript (lines 153-154). **---------------**


Reviewer #2: 

**In this study, the author has filled an important research gap by presenting a mathematical framework to define the sampling rates for confirmatory phenotypic testing so as to detect novel or previously uncommon resistance genotypes. By updating genotype-based diagnostics, the sensitivity of genotype-based antibiotic resistance should therefore maintained high. In addition, the authors also discussed multiple factors that require consideration when using the sampling model. Overall, it is well-structured and written.**
 **Please briefly describe where the datasets were collected from, hospital or other settings, country?**

This is now indicated in Table 1. **Line 61-63 As the cumulative sensitivity remains close to 1 in figure 1C and 1D, I am confused about "the genetic markers of resistance show decreased sensitivity over time". I guess the authors meant “For others, the original diagnostic genetic markers of resistance show ….”**

Thank you for pointing this out. We have now made this clarification. **Line 100 why the probability of having detected is 1 - X? when x was defined as the probability of new variant will be detected by time N in line 83-84.**

As the time to detection of the novel variant is derived by survival analysis, we are determining the time at which there is an x probability that we have failed to detect the novel variant (corresponding to a (1-x) probability that we have detected the variant). This is now clarified in lines 109-122.  **Line 101 Please provide details on how this equation was derived. Is it possible to provide an example like 90-92?**

We have now provided additional details on how this equation was derived (lines 109-122). We have further applied this metric (time to detection) to the calculation of surveillance cost effectiveness (lines 125-131). **Line 115 How was 3 and 15 additional cases calculated? Was it based on the equation in line 101.**

Yes, this calculation was based on the equation for the time to detection of the novel variant. However, we have now condensed this section and removed this calculation. **Figure 1A As the fractional incidence in GyrA S91F is a little bit higher than CIP NS in 2000, wondering why the sensitivity for GyrAS91F is 0.**

In 2000, there was a single reported CIP NS strain that did not have a GyrA S91F mutation. This could have been an MIC testing error, but the reported MIC of the strain was low (0.25 μg/mL) and may have been the result of a rare unknown resistance mechanism. In addition, that year two reported CIP S strains had the GyrA S91F mutation and ciprofloxacin MICs of 0.06 μg/mL. This MIC is just below the breakpoint, likely due to a lack of any additional mutations (*e.g.*, at GyrA-95, ParC-86, ParC-87, ParC-88, ParC-91) that are almost always identified together with GyrA S91F subsequently and associated with higher-level CIP resistance [1, 2]. **Figure 1F Is X = 0.95?**

Yes, in Fig. 2A (previously Fig. 1F), x = 0.95. This is now clarified in the figure legend.

---------------


Reviewer #3: 

**This is a very concise and thoughtful communication on some fundamental aspects of a new (and emerging) method for susceptibility testing. Although the theory is clear, I wonder whether real-life practice might not be (much) more complicated. Resistance can be based on a single genetic event (mutation or allele or gene) as in most of the examples put forward, but also on the combination of multiple events, such as for beta-lactam resistance in Enterobacteriales. For instance the single presence of OXA48 in Klebsiella may still render a susceptible phenotype for imipenem, but addition of any other beta-lactamase may render a non-susceptible phenotype (see Dautzenberg et al, Euro Surveillance 2014 Mar 6;19(9)). Not sure how this would influence the surveillance scheme (and if the authors could elaborate on this).**

Thank you for raising this issue. We agree that multifactorial resistance mechanisms will likely pose a challenge for genotype-based resistance diagnostics. We have now expanded our discussion of potential delays in diagnostic updates associated with identification of the genetic basis of novel resistance mechanisms (lines 225-226, 229-236).

---------------


Reviewer #4: 

Nicholas G. Davies, signed review

Review of: Surveillance to maintain the sensitivity of genotype-based antibiotic resistance diagnostics

**In this manuscript, the authors address an important question for managing antibiotic resistance: how much phenotypic testing for antibiotic resistance is needed to maintain the sensitivity of genotype-based assays for antibiotic resistance?

This is an interesting question with direct implications for policy. The manuscript is accompanied with well-chosen examples illustrating the problem of declining sensitivity of genotype-based diagnostics. There is also a good discussion of the context for the research and of considerations for putting suggestions into practice, as well as of alternative ways to maintain the sensitivity of diagnostics besides surveillance.**

**At the same time, the main result (line 87) is relatively straightforward to derive, which I think justifies a request that the authors go into a little more detail. For me, there is a slight disconnect here between the practical nature of the problem that is being addressed and the way in which the results are presented.

Specifically, I think the results could be rephrased (or elaborated) to be more relevant to policymakers. While it is interesting to know the required rate of testing, f, such that a new variant is detected with 100x per cent confidence by the time N variant cases have occurred (line 87), policymakers might be more interested in knowing the rate of testing f that maximizes the cost-effectiveness of surveillance, given the cost of testing, the cost of diagnostic failure, the sensitivity of the phenotypic assay for resistance (which may not be 100%, for example if there is a mixed infection), and so on.**

Thank you. We agree that it would be very useful to provide a framework for determining the phenotyping rate that maximizes cost-effectiveness (specifically, the rate of phenotyping that minimizes the cumulative cost of phenotyping and treatment failures). Unfortunately, it is likely very difficult to produce reliable estimates of the costs associated with treatment failures for a given pathogens, as these are obviously dependent on the ultimate infection outcome, are likely associated with a range of potential indirect costs in addition to the direct medical costs, may fail to reflect the potential cost associated with increased resistance due to inappropriate treatment, etc. Further, even using very general estimates for cost of treatment failure, relating the cost of phenotyping to the cost of treatment failures requires some knowledge of the rates at which novel variants emerge and grow in the population (in order to estimate the total cost of treatment failures for a given time period), which may be difficult to predict. However, we have now provided examples and discussion of how, given these parameters, one might use this model to calculate the rate of phenotyping that minimizes the cumulative cost of phenotyping and treatment failures (Figure 2B-C, lines 125-150 and 237-252).  **Similarly, I’m not sure the time before detection of a novel variant (line 101) is as interesting to policymakers as the expected number of diagnostic failures before detection. Also, as presented, this result depends upon a growth rate r which is probably quite difficult to predict from first principles—after all, we are talking about the relative fitness of novel mutations—and which would no longer be needed by the time it could be measured. Conversely, the number of failures before detection would not depend on r (assuming instantaneous testing).**

We agree that the time to detection on its own is not a practically useful or generalizable metric. However, this is a critical component in optimizing surveillance cost effectiveness as the cumulative cost of treatment failures and phenotyping associated with a given novel variant will be dependent on the time it takes for N cases to appear. Therefore, we have now applied this metric to the assessment of cost effectiveness. **I have made specific suggestions here but am open to alternatives—just suggesting more generally that the paper would be improved if the results were more directly translatable to decision-making.

Another potential issue that the manuscript doesn’t seem to address is that the model assumes that isolates subjected to phenotypic testing are selected randomly with respect to the overall population being monitored. But the model risks being overconfident if, for example, the relative rate of phenotypic testing varies spatially, since novel mutations will not in general be spread evenly through a population.**

We agree that it may be infeasible or unlikely to achieve random sampling across a population and that sampling bias may certainly impact the accuracy of the model. We have now added discussion of this point (lines 209-215) and are further currently working on a follow-up study to assess the impact of sampling bias on detection efficiency of novel variants. **Minor issues:

Lines 90-92: The meaning is clear, but there should be a statement about 95% confidence in this sentence.**

We have made this clarification. **Lines 98-102: It’s not quite clear from the way this is phrased whether a variant which is remaining at a stable frequency over time should have r = 0 or r = 1. Also, the probability of having detected the variant, which used to be x, now seems to be 1 – x, which is a little confusing. Finally, slightly more detail on how line 101 was derived would be clarifying.**

r is defined as the variant growth rate in the population (or the rate at which the variant is increasing in fractional incidence (and prevalence, assuming the overall case incidence remains constant) in a population. Therefore, if the variant is remaining at a stable prevalence, r would equal 0. We have now clarified the definition of r in the manuscript (lines 111-119). We have also now clarified that this metric was derived based survival analysis. Therefore, we are determining the time at which there is an x probability that we have failed to detect the novel variant (corresponding to a (1-x) probability that we have detected the variant). This is now clarified in lines 109-122. We have further clarified that r is defined as the variant growth rate **Line 175: the meaning of “identified as NAAT-negative” is a bit opaque—can this be rephrased?**

We have clarified the meaning of NAAT.

**References:**

1.	Vernel-Pauillac F, Hogan TR, Tapsall JW, Goarant C. Quinolone resistance in Neisseria gonorrhoeae: rapid genotyping of quinolone resistance-determining regions in gyrA and parC genes by melting curve analysis predicts susceptibility. Antimicrob Agents Chemother. 2009;53(3):1264-7. doi: 10.1128/AAC.01104-08. PubMed PMID: 19124663; PubMed Central PMCID: PMCPMC2650556.

2.	Grad YH, Harris SR, Kirkcaldy RD, Green AG, Marks DS, Bentley SD, et al. Genomic Epidemiology of Gonococcal Resistance to Extended-Spectrum Cephalosporins, Macrolides, and Fluoroquinolones in the United States, 2000-2013. J Infect Dis. 2016;214(10):1579-87. doi: 10.1093/infdis/jiw420. PubMed PMID: 27638945; PubMed Central PMCID: PMCPMC5091375.
